# Starch-based NP act as antigen delivery systems without immunomodulating effect

**DOI:** 10.1371/journal.pone.0272234

**Published:** 2022-07-29

**Authors:** François Fasquelle, Laurent Dubuquoy, Didier Betbeder

**Affiliations:** 1 Vaxinano, Loos, France; 2 Univ. Lille, Inserm, CHU Lille, U1286—INFINITE—Institute for Translational Research in Inflammation, Lille, France; Laurentian University, CANADA

## Abstract

The nasal route of immunization has become a real alternative to injections. It is indeed described as more efficient at inducing immune protection, since it initiates both mucosal and systemic immunity, thus protecting against both the infection itself and the transmission of pathogens by the host. However, the use of immunomodulators should be limited since they induce inflammation. Here we investigated *in vitro* the mechanisms underlying the enhancement of antigen immunogenicity by starch nanoparticles (NPL) delivery systems in H292 epithelial cells, as well as the NPL’s immunomodulatory effect. We observed that NPL had no intrinsic immunomodulatory effect but enhanced the immunogenicity of an *E*. *coli* lysate (Ag) merely by increasing its intracellular delivery. Moreover, we demonstrated the importance of the NPL density on their efficiency by comparing reticulated (NPL) and non-reticulated particles (NPL·NR). These results show that an efficient delivery system is sufficient to induce a mucosal immune response without the use of immunomodulators.

## 1. Introduction

The nasal route of immunization has now become a reliable alternative to injectable vaccine administration. This route of administration is much more convenient for patients and medical staff, and hence more suitable for mass vaccination, of particular pertinence during a pandemic such as the ongoing Covid-19 [1]. Moreover, the protection conferred via nasal administration is more efficient at inducing an immune protection than classical parenteral routes, as it initiates both mucosal and systemic immunity via a combination of humoral and cellular response [2, 3].

Vaccines currently designed for nasal immunization are generally made up with subunit antigens [4]. These preparations display a higher stability, better-defined manufacturing procedures and a lower toxicity compared to live attenuated vaccines, although being far less immunogenic. For this reason, they are often supplemented with adjuvants, whose function is to enhance both their recognition by antigen presenting cells (APC) and the subsequent activation of T and B cells [5, 6]. Adjuvants are generally classified into three categories according to their mechanism of action, namely immunomodulators, delivery systems or their combination.

Immunomodulators act by harmonizing the response of the immune system towards the antigen. These molecules often derive from pathogens, as in the case of soluble bacterial toxins (e.g. cholera toxins from *Vibrio cholerae*), nucleic acids (e.g. bacterial CpG ODN, viral dsRNA) or purified proteins and glycolipids (e.g. the flagellin or the LPS subunit monophosphoryl lipid A (MPL)), or else from other natural and synthetic sources (e.g. saponin QS-21 from *Quillaja saponaria* or the synthetic imidazoquinolines) [7, 8]. Most of them are recognized by APC via different membrane-bound and intracellular pattern recognition receptors (PRR), triggering the activation of pro-inflammatory intracellular pathways, finally leading to the promotion of either Th1 or Th2 cell population expansion [9]. However these advantageous mechanisms are of limited value for nasal administrations since the inflammation they induce could lead to serious adverse effects [10–12].

On the other hand, nanoparticle (NP) delivery systems for antigens are promising strategies to enhance the nasal immunogenicity of subunit vaccines. Indeed, the encapsulation of antigens prolongs their mucosal residence time through enhanced interactions at mucosal level and protection from proteolysis [13], and can also increase the intracellular delivery into epithelial cells and APC, thereby inducing a greater immune response [14]. These systems include lipid-based particles (e.g. liposomes, micelles, emulsions), polymeric- and polysaccharidic-based vectors (e.g. poly-lysine, starch, polylactic-co-glycolic acid (PLGA), chitosan) or inorganic materials (e.g. gold and mesoporous silica), which can be loaded with the antigens within their core or on their surface [15].

In addition, some delivery systems, on account of their inner composition, are also able to modulate the immunity by targeting TLR or inducing inflammation. As an example, the AS01 adjuvant used in the injected licensed malaria vaccine Mosquirix^TM^, is an immunostimulating liposomal carrier containing MPL and QS-21. AS01 can encapsulate antigens and simultaneously stimulate APC through TLR4 targeting, leading to Th1 mediated immune response [16, 17]. Similarly, the immune stimulating complexes ISCOMs and ISCOMATRIX are liposomes made from phospholipids, cholesterol and saponins, and trigger Th1 and Th2 responses with potential applications in mucosal and injectable immunization [18, 19]. In the same way, the oil-in-water emulsion AS02 composed of saponins and MPL carries antigens and triggers Th1 immune response through TLR4 recognition [20, 21].

While these carriers can trigger the immunity, their use for nasal administration of vaccines still raises concerns and safety issues due to their local reactogenicity.

Importantly, the design of NPs is crucial to ensure an efficient antigen association and intracellular delivery. While it is well documented that the size and surface charge of the delivery system can greatly improve the nasal immunization, the importance of the NPs density has not yet been studied [22].

Cationic reticulated maltodextrin-based nanoparticles with a phospholipid core (NPL) are porous and spherical NPs, able to be loaded with and deliver a large amount of antigen into epithelial and immune cells [23, 24]. Due to their anionic lipid core, NPL penetrate the mucus after nasal administration and therefore increase the antigen retention time in the nasal mucosa [25–27]. Accordingly, they are ideal for nasal immunization [28–30].

In the present study, we investigated the mechanisms by which NPL enhance the immunogenicity of antigens (here an *E*. *coli* whole lysate (Ag)) toward the nasal mucosa. Moreover, we studied the importance of NPL density on their efficiency by comparing reticulated (NPL) and non-reticulated particles (NPL·NR). The absence of reticulation did not vary the size nor the surface charge, therefore allowing the subsequent comparisons. The ability to load the Ag was assessed by PAGE analysis. In addition, the uptake and antigen delivery were evaluated by flow cytometry. Finally, the ability to enhance the Ag immunogenicity was evaluated by measuring the cytokine and chemokine secretions of airway epithelial cells.

## 2. Material and methods

### 2.1. Material

Maltodextrin was purchased from Roquette (France) and DPPG (1,2-dipalmitoyl-snglycero-3-phosphatidylglycerol) was from Lipoid (Germany). Cell culture media (RPMI 1640, DMEM and IMDM), fetal calf serum (FCS), nonessential amino acids, TrypLE™ Express (0,45 mg/mL EDTA), L-glutamine, trypan Blue 0.4%, phosphate buffered saline (PBS), NaOH, ethanol, DiI (1,10-dioctadecyl-3,3,30,30-tetramethylene), fluorescein isothiocyanate (FITC), DQ™ Green BSA, Slide-A-Lyzer™ Dialysis Cassettes 10MWCO and Micro BCA protein assay kit were all purchased from ThermoFisher Scientific (France). Epichlorohydrin (1-chloro-2,3-epoxypropane), GTMA (glycidyl-trimethyl-ammonium chloride), NaBH4, bovine serum albumin (BSA), purified bovine submaxillary mucins and PD-10 Sephadex desalting column were purchased from Sigma-Aldrich (France). Human TNF-α, IL-1ß, IL-6 and IL-12p40 ELISA Kit were purchased from Biolegend (San Diego, CA, USA) and the cytokine multiplex assay was purchased from Bio-Techne (France). The *E*. *coli* whole lysate (Ag) was obtained by isopropanol inactivation of an O78:K80 strain and supplied by Eurofins Bactup (France). The human lung epithelial carcinoma cell line NCI-H292 [H292], ATCC CRL-1848 (hereafter H292) and the human monocyte cell line THP-1, ATCC TIB-202 (hereafter THP-1) were purchased from ATCC (United Kingdom).

### 2.2. NPs syntheses and labeling

#### 2.2.1. Maltodextrin nanoparticles

NPL were synthesized as previously described [31]. Maltodextrin (100 g) was dissolved in 2 N sodium hydroxide with magnetic stirring at 25°C, and then epichlorohydrin (4.72 mL) and GTMA (31.08 g) were added to obtain a cationic polysaccharide gel. The gel was then neutralized with acetic acid and crushed using a high-pressure homogenizer (LM20, Microfluidics, France). The particles obtained were then purified by tangential flow ultra-filtration (AKTA flux 6, GE Healthcare, France) using a 750 kDa membrane (GE Healthcare, France) to obtain purified cationic particles (NP^+^). These NP^+^ were mixed thereafter with 70% DPPG (% weight) at 80°C for 2 h and filtered through a 0.2 μm filter to produce NPL.

Less dense NPL were produced as above but without epichlorohydrin, to avoid the reticulation of the maltodextrin. The non-reticulated NP^+^ (NP^+^·NR) were also mixed with 70% DPPG (% weight) to obtain non-reticulated NPL·NR.

#### 2.2.2. Nanoparticle labeling

NPL and NPL·NR were labeled with DiI by mixing 1% w/w dye (1 mg/ml in ethanol) with the particle suspension overnight. Each labeled particle was purified by dialysis using a 10 kDa dialysis cassette to remove the unbound dye, and the labelling was confirmed by fluorometry (Fluoroskan Ascent, Thermo Scientific, France).

### 2.3. Nanoparticle and formulation characterizations

#### 2.3.1. Dynamic Light Scattering (DLS)

The average hydrodynamic diameters were measured in 23 mM NaCl and at 25°C by DLS using a Zetasizer Nano ZS (Malvern Instrument, France). The results are expressed as the mean Number ± standard deviation (sd) of at least three independent syntheses.

#### 2.3.2. Zeta potential

Zeta potentials were measured in ultrapure water and at 25°C by electrophoretic light scattering (ELS) using a Zetasizer nanoZS. The results are expressed as the mean ± sd of at least three independent syntheses.

#### 2.3.3. NPs’ density

The NPs’ maltodextrin-density was evaluated by liquid pycnometry [32]: the weight of 10 mL NP^+^ and NP^+^-NR solutions (5%) were compared to 50 mg of freeze-dried NPs and ultrapure water. For each NP, the solution’s densities were then calculated on 3 independent batch of synthesis, and with 10 measurement per batches, as:

$$\rho (NP)=\frac{m(NP\;lyoph).\rho (H2O)}{m(H2O)-(m(NP\;sol)-m(NP\;lyoph))}$$

### 2.4. Protein labelling

An Ag suspension (5 mg/mL) was mixed with FITC (1% w/w) in a 0.1 M carbonate/bicarbonate buffer (pH = 8.3) with magnetic stirring at room temperature for 1 h. Ag-FITC was purified from the unbound dye by dialysis in 10 kDa dialysis cassettes. The labelling was confirmed by fluorometry (Fluoroskan Ascent, Thermo Scientific, France), and the final protein concentration was measured using microBCA assay, according to the manufacturer’s instructions.

### 2.5. Post-loading of antigens within nanoparticles

The loading of NPL and NPL·NR with the Ag was performed by mixing the components in ultrapure water at room temperature for at least 1 h, at different protein/NPL weight %.

The protein association was evaluated with native polyacrylamide gel electrophoresis (native PAGE): the formulations were supplemented with a non-denaturing buffer (Tris–HCL 125 mm (pH 6.8), 10% glycerol and 0.06% bromophenol blue) and run on a 10% acrylamide-bisacrylamide gels for 15 min at 120 V then 45 min at 180 mV. Under these conditions, NPs are too large to enter the gel and NP-associated proteins cannot migrate either, while unassociated proteins migrate normally. The gels were then stained by the silver nitrate method, scanned and the protein strains quantified using the ImageJ software [24].

### 2.6. OVA-DQ hydrolysis in presence of proteases

To evaluate the ability of NPL and NPL·NR to protect antigens from proteolysis, 3.75 μg of OVA-DQ was associated with 11.25 μg NPL or NPL·NR (30% weight ratio), in 200 mL of PBS (150 mM; pH 7.2), in a 96 well plate. Free OVA-DQ was used as negative control. For trypsin treatment, 50 μL of 5 trypsin units diluted in PBS was added in each well, for 0.84 μM final concentration of enzyme, and the plate was incubated for 240 minutes at 37°C. The proteolysis was observed by the generation of OVA-DQ fluorescent fragments, measured by fluorometry (λex: 485 nm, λem: 527 nm), with the 100% of degradation set over the protein alone.

### 2.7. Cell culture and assays

Human H292 epithelial cells were maintained in RPMI 1640, supplemented with 10% heat-inactivated FCS, 100 U/mL Penicillin, 100 mg/mL streptomycin and 1% (v/v) L-glutamine at 37°C in a humidified, 5% CO2 atmosphere.

Protocols concerning the culture, differentiation and experiments made with THP-1 cells are presented in Supplementary data.

#### 2.7.1. NPs endocytosis

H292 cells were seeded in 24-well plates at a density of 5 x 10^4^ cells per well until confluence. They were then treated for 5 to 180 min with 9 μg DiI-labeled NPs in 500 μL of fresh medium.

Cells were harvested with TrypLE™ Express, collected by centrifugation and diluted in PBS before cytometry analysis on an Attune^TM^ NxT (ThermoFisher Scientific). Isolated cells were selected by their size and cellular complexity with a gating on SSC-A:FSC-A then SSC-A:SSC-H, before measuring the percentage of DiI-positive cells and mean fluorescence intensities (MFI). Analyses were made on 7,500 individual cells and the results are expressed as the mean ± sd of at least three independent experiments.

#### 2.7.2. Intracellular protein delivery by NPs

Cells were seeded in 24-well plates at a density of 5 x 10^4^ cells per well until confluence. They were then treated for 30 min in 500 μL of fresh medium with 3 μg Ag-FITC, alone or encapsulated in NPL or NPL·NR.

Cells were harvested with TrypLE™ Express, collected by centrifugation and diluted in PBS (without calcium or magnesium) before cytometry analysis on an Attune^TM^ NxT (ThermoFisher Scientific). Trypan blue (40 μg/mL) was added to quench FITC extracellular fluorescence [33]. Isolated cells were selected by their size and cellular complexity with a gating on SSC-A:FSC-A then SSC-A:SSC-H, before measuring the percentage of FITC-positive cells and MFI. Analyses were made on 7,500 individual cells and the results are expressed as the mean ± sd of at least 3 independent experiments.

#### 2.7.3. In vitro evaluation of epithelial cell immune activation

Cells were seeded in 96-well plates at a density of 10^4^ cells per well until confluence. They were then treated for 24 h, in 250 μL, under different conditions: RPMI (as negative control), 1μg/mL LPS (as positive control), 15 μg/mL empty NPL or NPL·NR (*i*.*e* without Ag), 5 μg/mL Ag alone, and the formulations of Ag encapsulated in each NP (5 μg/mL Ag + 15 μg/mL NP). Cell culture supernatants were collected and stored at -20°C. Cytokine production was measured with a multiplex assay and read with a Bio Plex 100 multiplex system (BioRad, France).

### 2.8. Statistical analysis

Multigroup comparisons were analyzed by one-way Anova followed by the Tukey test, or two-way Anova followed by the Sidak test. Comparisons between 2 groups (n > 30) were analyzed by two-tailed Student’s t test for unpaired data.

A *p* value < 0.05 was considered to be statistically significant. Data were analyzed with Prism Software (GraphPad Software Inc. 8.4.2).

## 3. Results and discussion

### 3.1. Characterization of NPL with and without reticulation

**Fig 1:**
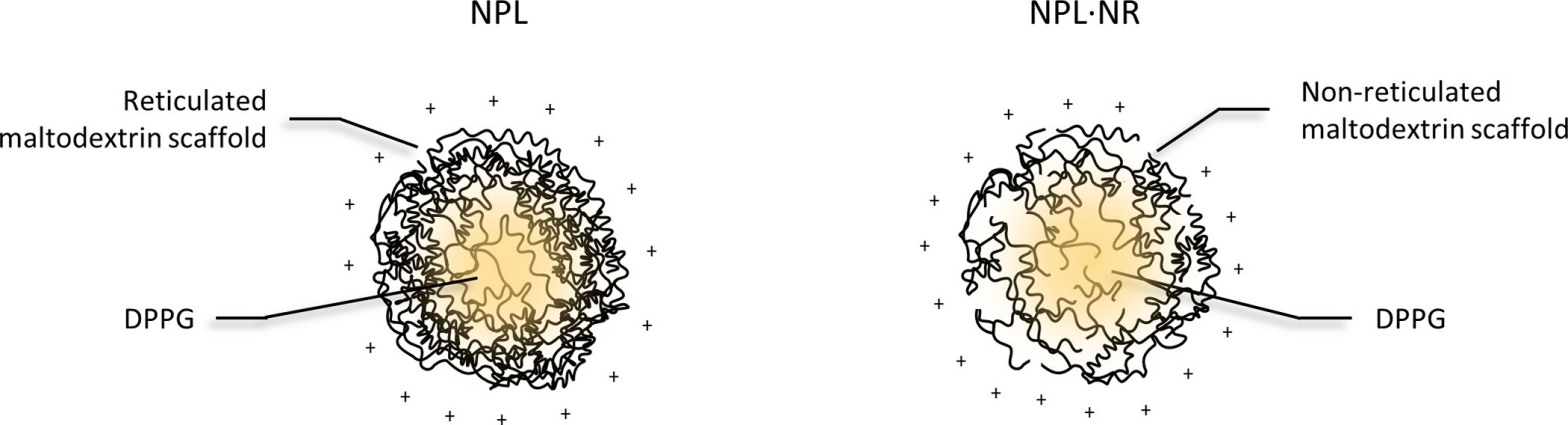
Schematic representation of NPL (cationic particles with reticulated maltodextrin and a DPPG phospholipid core) and NPL·NR (cationic particles with non-reticulated maltodextrin and a DPPG phospholipid core).

NPL with different densities were synthesized: NPL are reticulated cationized maltodextrin NPs with a lipid core, and NPL with a non-reticulated maltodextrin were synthesized as well to obtain less dense NPL (NPL·NR, Fig 1). Their density was measured by pycnometry. NPL showed a maltodextrin density of 1.37 g/cm^3^ and NPL·NR of 1.21 g/cm^3^ (*p*<0.01, Student *t-test*), confirming that the reticulation increased the maltodextrin density (Table 1). Indeed, maltodextrin is known to naturally produce microparticles when diluted in water, due to hydrophilic and hydrophobic interactions between the polysaccharides chains [34, 35]. Without a crossing agent their homogenization led to the formation of nanoparticles, less dense than the reticulated ones. Importantly, the absence of reticulation may affect other parameters such as the NPs’ elasticity or their porosity.

**Table 1:** Nanoparticles’ characterization.

	HYDRODYNAMIC DIAMETER (NM)	PDI	Z-POTENTIAL (MV)	MALTODEXTRIN DENSITY (G/CM^3^)
**NPL**	35 ± 2	0.28	+ 38 ± 1	1.37 ± 0.063
**NPL·NR**	24 ± 3	0.25	+ 37 ± 1	1.21 ± 0.039

The diameters and surface charges were measured in water, by DLS (in number) and ELS, respectively, and their density by liquid pycnometry. Results represent the mean ± SEM of at least three independent measurements, made on three independent batches.

NPL had a diameter of 35 ± 2 nm (mean ± sd), and their surface charge was cationic with a zeta-potential of +38 ± 1 mV. No variation of size nor zeta potential was observed between NP^+^ and NPL, corroborating our previous experiments showing that the anionic phospholipids were indeed inside the particles (S1 Table) [36]. Without reticulation, they had a diameter of 24 ± 3 nm, and a cationic surface of +37 ± 1 mV. This suggests that the lowered density did not affect the particles’ size nor their surface charge. This size and cationic surface charge is well described as being more efficient for cell delivery [37, 38]. Moreover, no significant variation of diameter nor surface charge was observed between NP^+^·NR and NPL·NR, confirming that even without reticulation, the phospholipids were also unequivocaly encapsulated within the particles (S1 Table).

### 3.2. Impact of the density on NPL protein loading

**Fig 2:**
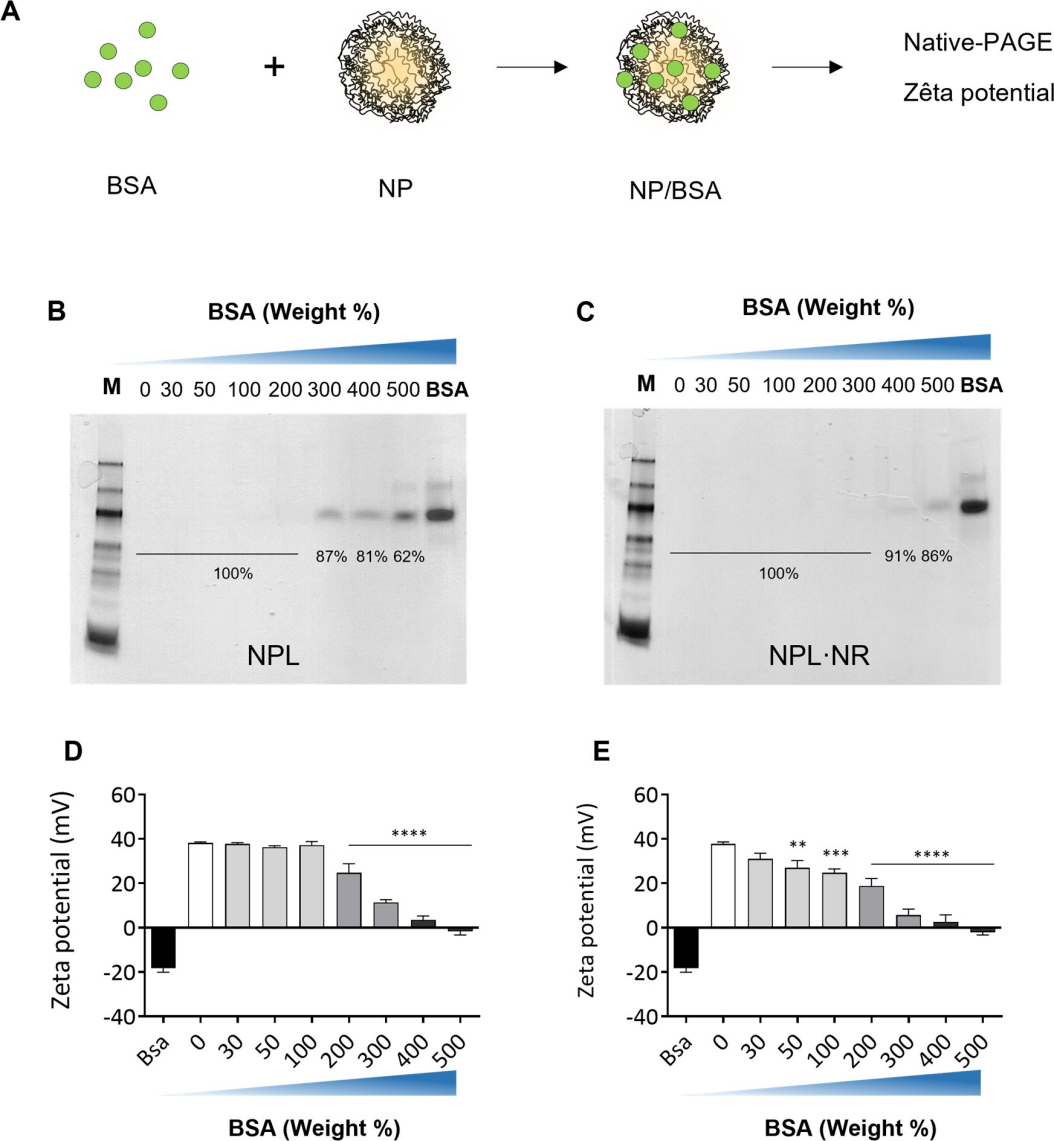
The NPs were loaded with increasing amount of BSA (A). The loading efficiency of NPL (B) and NPL·NR (C) was evaluated by native polyacrylamide gel electrophoresis. The internal or surface association to NPL (D) and NPL·NR (E) was evaluated by zeta-potential measurements, and the results represent the mean ± SEM of at least three independent measurements. Statistical analysis was performed by one-way ANOVA ** p < 0.01, *** p < 0.001, **** p < 0.0001 by One-way ANOVA.

To evaluate the influence of the particles’ density on the protein encapsulation, NPL and NPL**∙**NR were loaded with increasing amounts of albumin. The loading efficiency was measured by Native PAGE, and the variation of the surface charge by ELS (Fig 2A).

The electrophoresis showed that the maximum BSA loading was 200% for NPL (Fig 2B, % weight), and 300% without reticulation (Fig 2C, % weight), suggesting that the protein loading reached saturation more quickly for the denser NPs. Hence, reticulation may lead to tighter pores in the NPL maltodextrin scaffold compared with NPL**∙**NR, thereby decreasing the available entry for loading the proteins. At the same time, however, the total number of NPL·NR was higher than that of NPL for the same mass owing to their lower density, which may also have facilitated the antigen loading expressed in weight/weight.

In parallel, the NPLs’ surface charge remained constant from 30% to 100% BSA (+38 mV) and dropped when loaded with 200% or more protein (+19 mV). This indicated that BSA was encapsulated inside the particles from 30% to 100%, and thereafter the saturation of the inner core, resulted in the surface loading of protein from 200% and above. On the other hand, the absence of reticulation lead to a continuously decreasing surface charge when loaded with 30% BSA and more, indicating that the protein was mostly bound at the NPs’ surface, hiding cationic charges. Interestingly, this variation of surface charge may influence the antigen’s bioavailability: NPs with cationic surfaces interact more easily with cell membrane than neutral or anionic NPs, while a surface association could also influence the antigen’s presentation and the efficiency of its delivery inside mucosal cells [24].

### 3.3. Influence of NP density on albumin hydrolysis

**Fig 3:**
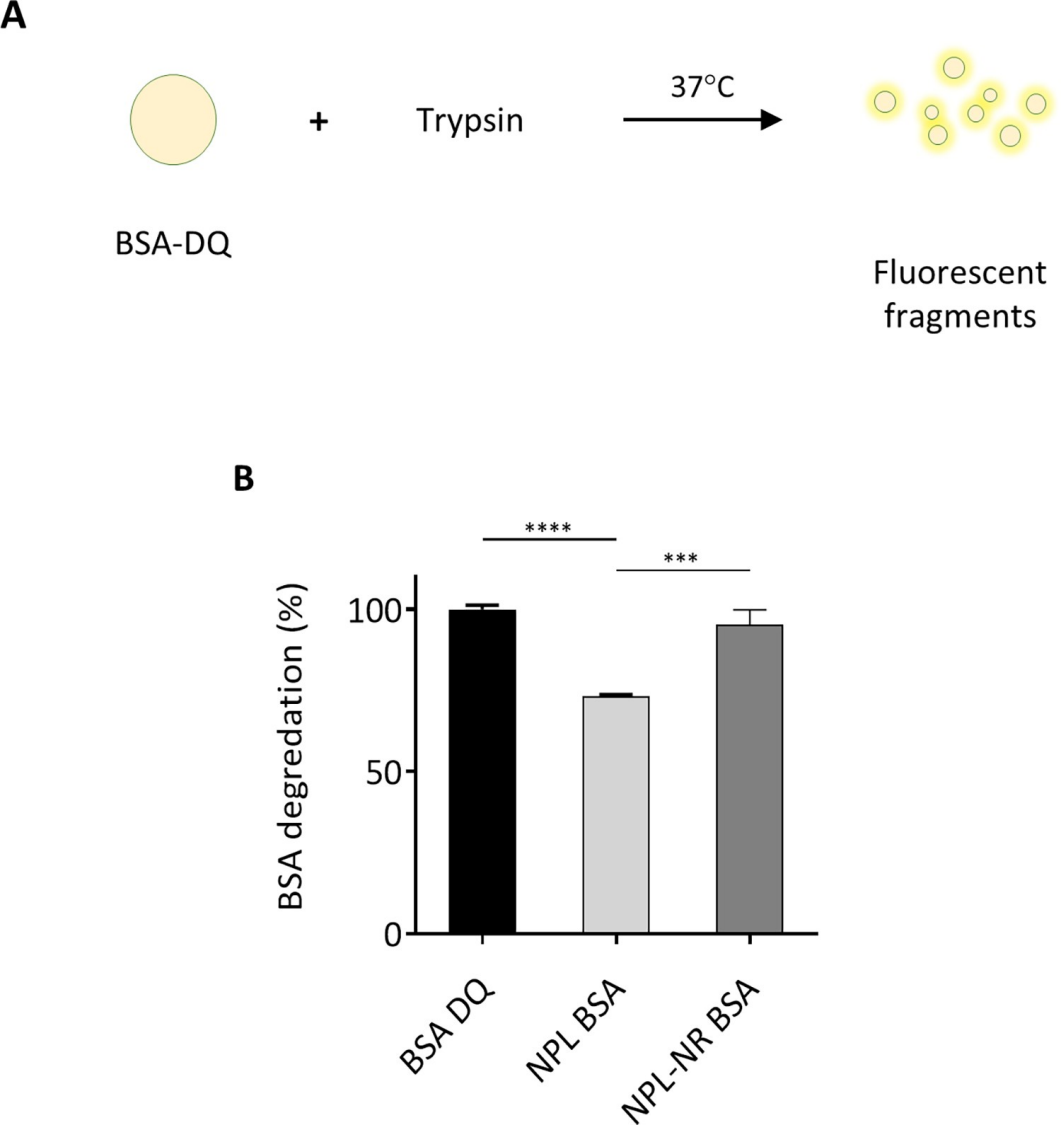
BSA-DQ alone or loaded in NPs was incubated in the presence of trypsin, for 2 h at 37°C and pH 7.2 (A). The proteolysis was measured by fluorometry (B). The results represent the normalized mean ± SEM of 3 independent experiments, and statistical comparisons were made by one-way ANOVA. * p < 0.05, ** p < 0.01, **** p < 0.0001.

Delivery systems can enhance an antigen’s bioavailability by protecting it from local proteolysis, a factor of particular concern in the enzymatic environment of the nasal mucosa. Therefore, to study the influence of the NPs’ density on the preservation of the antigen, BSA-DQ was associated with NPL and NPL·NR for 1h and placed in solution with trypsin (Fig 3A). The fluorescence intensity of BSA-DQ hydrolysis was measured for 2 h at 37°C and pH 7.2.

When associated with NPL, 73% of BSA proteolysis was observed after 2 h, while without reticulation 95% protein degradation was observed after 2h (Fig 3B). Therefore, NPL significantly protected the antigen from proteolysis. In contrast, no protection was observed when the protein was free or associated with non-reticulated NPL. This difference is probably due to the greater protein exposure on the reticulated NPs’ surface, as described above (Fig 2D and 2E).

### 3.4. NPs’ endocytosis

**Fig 4:**
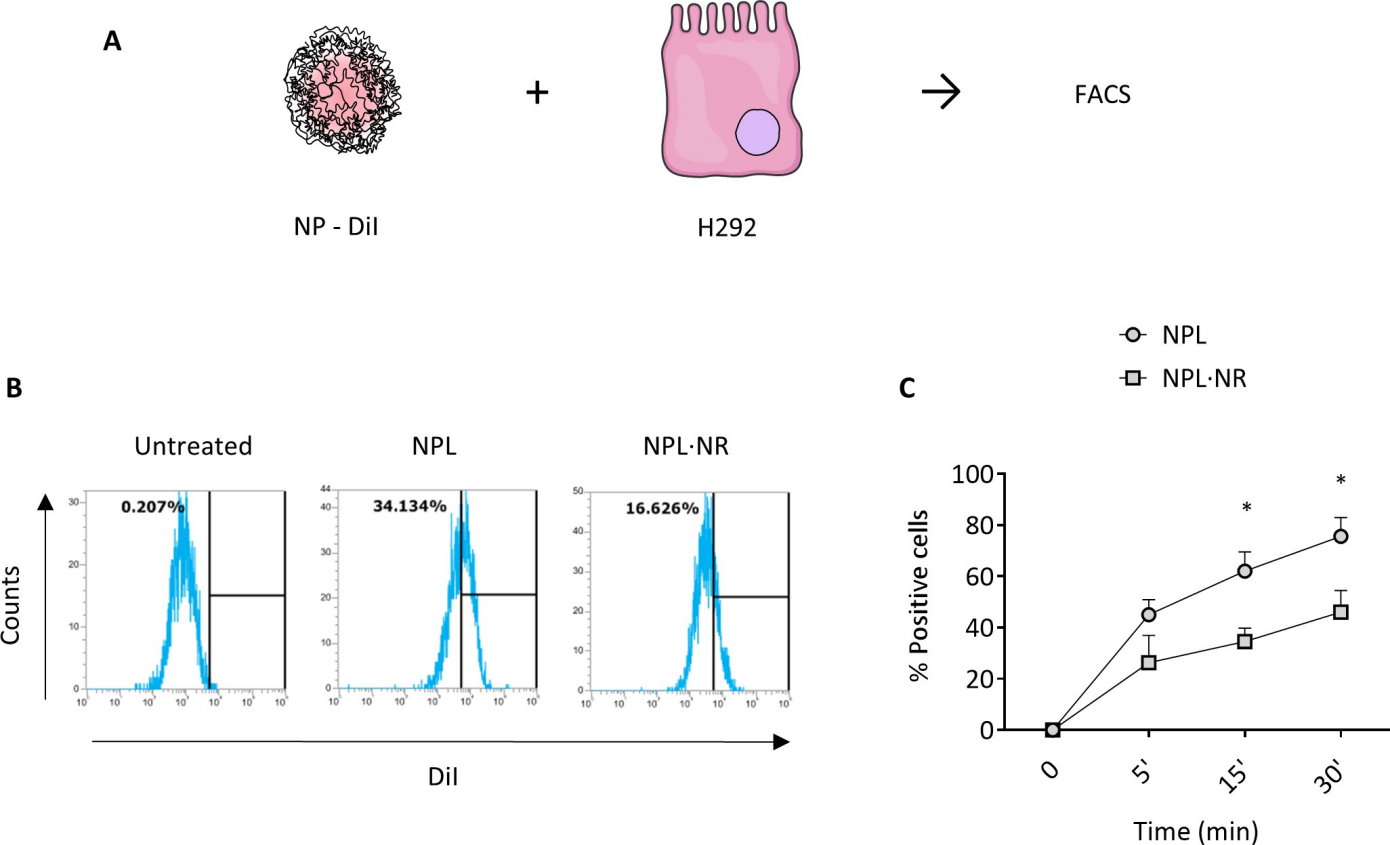
DiI-NPs were incubated with epithelial cells for 5 to 30 minutes (A). Their endocytosis on H292 cells was evaluated by flow cytometry (B & C). The results represent the mean ± SEM of at least 3 independent experiments, and the statistical comparison were made by ANOVA tests. ** p < 0.01, *** p < 0.001.

DiI-labeled NPL and NPL·NR endocytosis were evaluated on airway H292 epithelial cells by flow cytometry (Fig 4A & 4B).

Owing to their size and cationic surface charge, the particles were rapidly taken up by the cells after only 5 minutes of incubation (Fig 3A and 3B). NPL exhibited more rapid endocytosis, suggesting that denser particles interact more easily with the cell membrane than the less-dense ones. Indeed, for particles of diameter < 50 nm, pinocytosis is the main route of endocytosis, even for phagocytic cells, due to the formation of membrane invaginations which are easily triggered with stiff particles [39]. Similarly, NPL were taken up faster by THP-1-differentiated macrophages, but no difference was observed between the two NPs for THP-1-differentiated dendritic cells (S1 Fig).

Similar results were obtained previously, with (i) liposome NPs, where an increased density increased the phagocytosis by APC [40]; or with (ii) PEG NPs, where the rigid particles were taken up to a greater extent by mammal gland epithelial cells and macrophages than the less-dense, softer ones [41].

These results confirm that the NPs’ density is an important parameter determining their endocytosis by airway epithelial cells.

### 3.5. Ag loading and cell delivery

**Fig 5:**
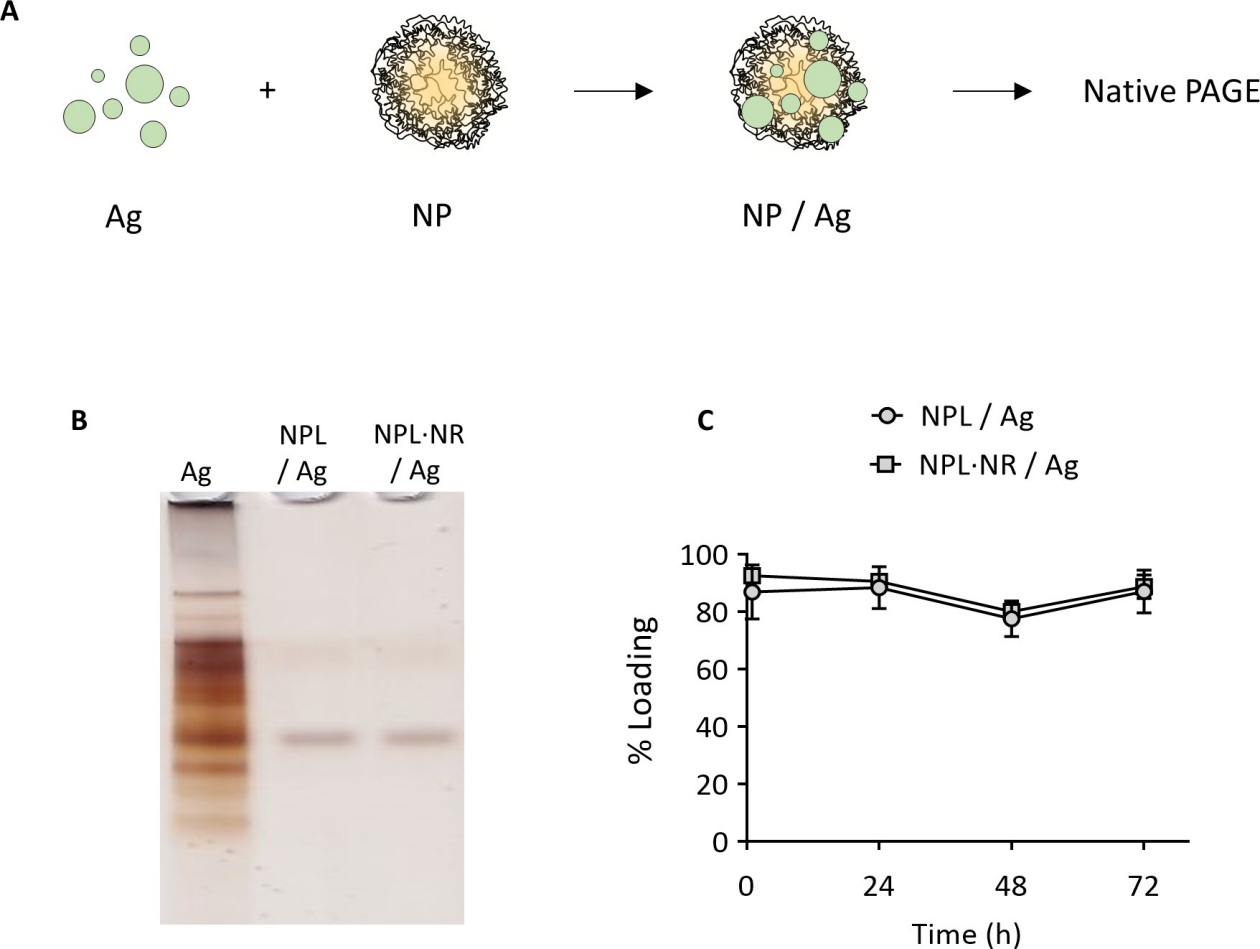
A: Schematical representation of the NPs/Ag formulations. B: Native PAGE of NPs/Ag (30% weight ratio) after 1h; C: Loading stability of the NPs/Ag (30% weight ratio) for 3 days.

The ability of NPL and NPL·NR to be loaded with Ag was evaluated (Fig 5). After 1 h, the particles had been loaded with the majority of the antigen, regardless of their density (87 ± 9% association for the NPL and 93 ± 2% for the NPL·NR). After 72 h, no protein release was observed. NPs were thus able to keep the antigens loaded over time, with no implication of the maltodextrin’s reticulation.

**Fig 6:**
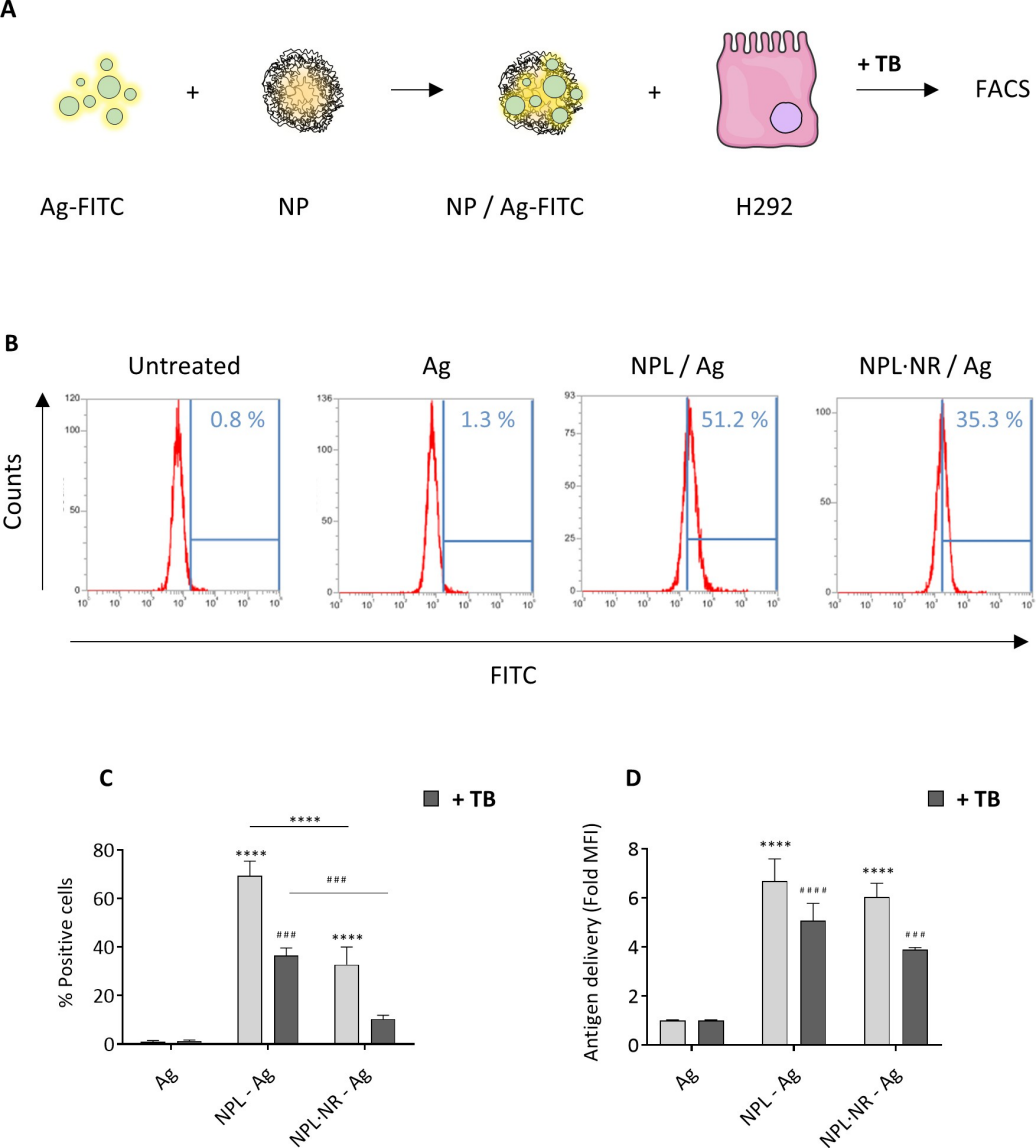
NPs were loaded with FITC-labeled Ag (30% weight ratio). The Ag delivery in H292 cells was evaluated in absence and presence of trypan blue (TB) by flow cytometry (A). The percentage of positive cells (B and C) and the relative amount of antigen (C) are presented. The results represent the mean ± SEM of at least 3 independent experiments, and the statistical analyses were performed by two-way ANOVA tests. Stars represent statistical differences for the values without TB, and hash symbols for the values with TB. * p < 0.05, ** p < 0.01, *** p < 0.001.

Then Ag-FITC delivery was evaluated by flow cytometry on H292 cells after 30 minutes incubation (Fig 6A). Trypan blue (TB) was used to quench membrane-bound fluorescence. By this process, the overall Ag cell interaction, including membrane-bound antigens, was measured [33].

The Ag’s total cell association was significantly greater when associated with NPs, regardless of their density, compared to free Ag (Fig 6B–6D). This cell association was also greater with NPL than with NPL·NR. Moreover, in the presence of TB, we observed that the intracellular delivery was also significantly greater with NPL than NPL·NR. These results are in accordance with the NP endocytosis experiments, confirming that the greater the particles’ density, the greater their intracellular uptake. Moreover, as demonstrated above, NPLs’ surface charge remains cationic in presence of antigens thanks to their reticulation. Consequently, formulations made with dense NPs are more likely to interact with the cell membrane and to be endocytosed. Similar results were observed on macrophages and dendritic cells (S2 Fig).

To our knowledge, these results are the first to clearly demonstrate the importance of the NPs’ rigidity for the efficacy of intracellular antigen delivery.

Nevertheless, the efficiency of delivery is insufficient to evaluate a formulation’s adjuvanticity, and its immunological activation still needed to be assessed. As H292 cells are known to express both membrane and intracellular PRR, the immune activation would be expected to be more intense thanks to the delivery system [42, 43].

### 3.6. Formulation’s immunogenicity on epithelial cells

As the first physical and immune barrier in the mucosa, epithelial cells are sensitive to various exogenous stimulations such as pathogenic/antigenic patterns. If need be, they will trigger the innate immune response by secreting a cocktail of cytokines and chemokines, which may in turn initiate the adaptative immunity cascade [44].

Paradoxically, the addition of adjuvants or immunomodulators in mucosal vaccines may *a fortiori* favorize the detection of the antigen (either via PRR targeting or via the induction of local inflammation), but will often lead to side-effects due to the high mucosal reactogenicity. Consequently, the development of non-inflammatory delivery systems is crucial for mucosal routes of administration [45].

**Fig 7:**
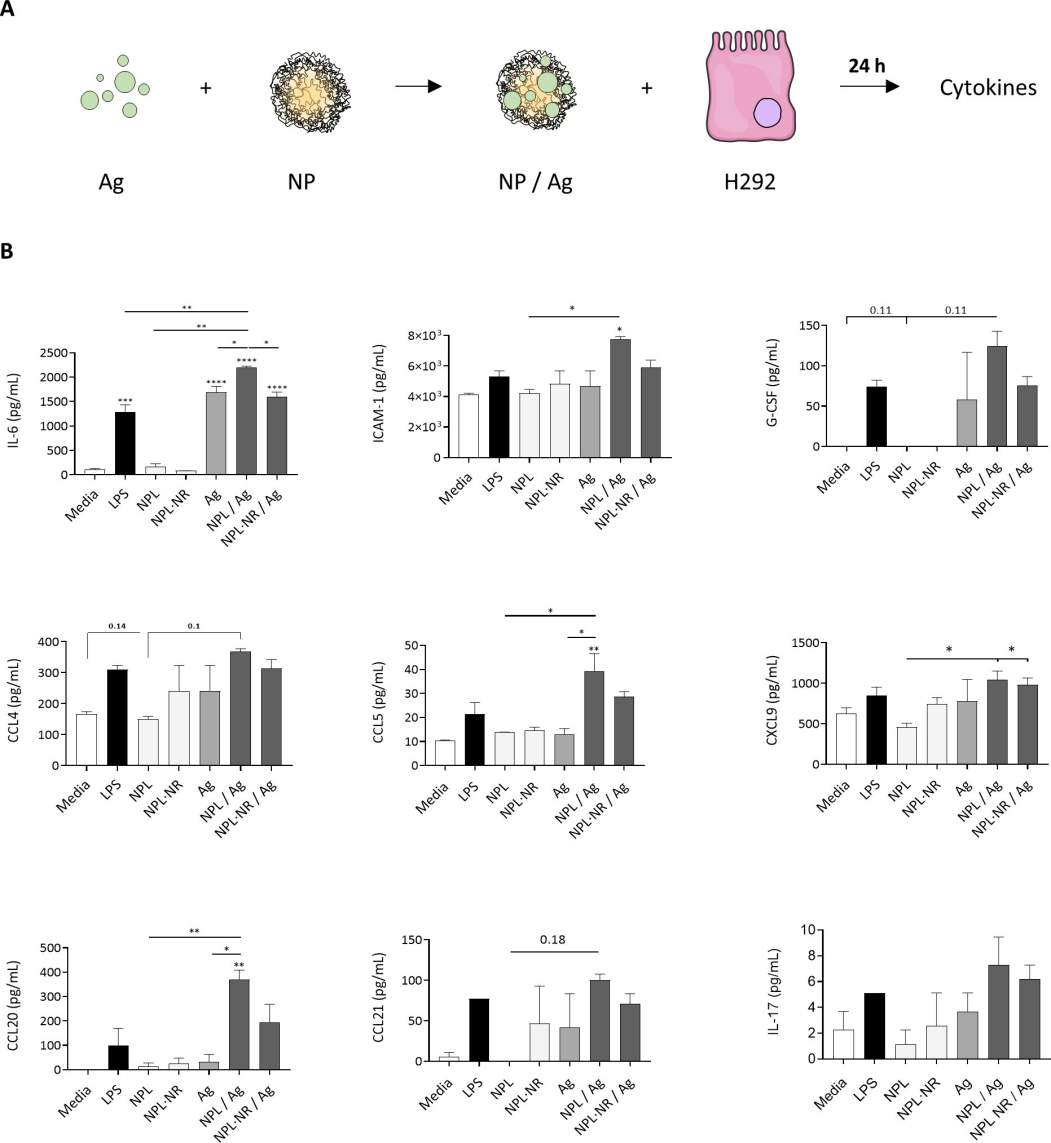
The immunogenicity of NPs and Ag was evaluated on H292. The cells were incubated for 24h with LPS (1 μg/mL), empty NPs (i.e without Ag, 15 μg/mL), Ag (5 μg/mL) alone or Ag loaded into NPs (30% weight ratio), and the cytokine and chemokine secretions were measured by Multiplex assay. Results represent mean ± SEM of at least 3 independent experiments, and statistical comparisons were made by one-way ANOVA. * p < 0.05, ** p < 0.01, *** p < 0.001 **** p <0.0001.

We showed in a previous study that NPL did not induce plasma membrane damage nor mitochondrial dysfunction in epithelial cells, even at high doses [46]. Here, we incubated airway epithelial cells with empty NPs, free Ag, and Ag loaded in NPs. We also measured the secretion of pro-inflammatory cytokines and chemokines as a function of the NPLs’ density (Fig 7A).

After 24h incubation with empty NP, no chemokine or pro-inflammatory cytokine secretion was observed, regardless of their density (Fig 7B). Thus, their uptake did not trigger any immune signaling pathways, nor any inflammation. This is in opposition to other immunomodulating mucosal adjuvants and delivery systems, which directly trigger the immune response by themselves [47].

Furthermore, free Ag was only able to stimulate the secretion of pro-inflammatory IL-6 (1685 pg/mL) and G-CSF (58 pg/mL). This confirms its innate recognition by epithelial cells PRR, but also its inability to fully activate the epithelial immune response, despite the breadth of the antigen’s composition.

However, when encapsulated in the NPL, the Ag stimulated to a much higher extent the secretions of various immune messengers responsible for the establishment of both innate and adaptative responses: IL-6 (2200 pg/mL) which is a pro-inflammatory cytokine known to stimulate the production of chemokines, and to promote B cell differentiation and T cell proliferation [48]; ICAM-1 (7750 pg/mL) which enhance the adhesion of leukocyte among which neutrophils with epithelial cells [49]; G-CSF (125 pg/mL), known to influence dendritic cell and T cell function to a Th2 phenotype [50]; and the chemokines CCL4 (368 pg/mL), CCL5 (39 pg/mL), CXCL9 (1043 pg/mL), CCL20 (370 pg/mL), CCL21 (100 pg/mL), known to promote the local recruitment and infiltration of macrophages, dendritic cells, innate and memory B/T cells [p. 20, 51–55]. It also seems like NPL/Ag formulations trigger the secretion of IL-17 more than the antigens, but no statistical increase was measured. No variation of IL-8, IL-1β nor TNF-α secretion was detected. Moreover, for CCL20 and IL-6, the secretion was significantly greater than pure LPS (*p* < 0.05 and *p* < 0.01, respectively).

Thus, the Ag delivered by the NPL particles increased detection of the antigen by epithelial cells as well as their subsequent activation. As observed for the intracellular delivery studies, the formulation made with NPL was significantly more immunogenic than the NPL·NR formulation, confirming the importance of the NPs’ density for the vaccine’s efficiency.

In parallel, the particles’ adjuvanticity was also evaluated on macrophages and dendritic cells, by measuring the secretion of different cytokines (S3 Fig). The formulations made with NPL induced a 5-times increase in IL-1ß secretion by macrophages independent of their density, suggesting a greater pro-inflammatory activation. However, only the NPL formulations increased the IL-12p40 secretion, suggesting a Th1/Th17 orientation. The same pattern was observed for dendritic cells, where only the formulations made with the NPL increased the IL-1ß and the IL-12p40 secretions.

Altogether, these results suggest that NPL are able to enhance the antigen’s immunogenicity toward epithelial cells thanks to greater, more efficient antigen delivery.

## 4. Conclusion

In this study, we demonstrated that NPL only act as a delivery system without inducing inflammation or immunomodulation by themselves. The adjuvant effect was only visible here when delivery of Ag was efficient, leading to a strong activation of the epithelial cells via the secretion of cytokines and chemokines and promoting the local recruitment of DC and T-cells. The importance of the NPs’ density on this efficiency was also demonstrated, with denser, more rigid particles proving more efficient than less-dense, softer particles.

Since few nasal vaccines are authorized for administration in humans due to concern over significant side effects, despite the large number of promising studies on this vaccination route, the development of inert delivery systems should be encouraged to obviate the use of immunomodulating molecules and stimulate the discovery of safe and efficient nasal vaccines.

## Supporting information

S1 Methods THP-1 cell culture and assay. (DOCX)

S1 Table The diameters and surface charges were measured in water, by DLS (in number) and ELS, respectively. Results represent the mean ± SEM of at least ten independent measurements, made on three independent batches.
(DOCX)

S1 Fig NPs-DiI endocytosis on THP-1 differentiated macrophage (left) and immature dendritic cells (right) cells was evaluated by flow cytometry. The results represent the mean ± SEM of at least 3 independent experiments, and the statistical analysis were made by two-way ANOVA. * p < 0.05.
(TIF)

S2 Fig Ag was labeled with FITC and loaded in NPL and NPL·NR. Its endocytosis on THP-1 differentiated macrophage (A) and immature dendritic cells (B) was evaluated by flow cytometry. The results represent the mean ± SEM of at least 3 independent experiments, and the statistical analysis were made by two-way ANOVA tests. * p < 0.05.
(TIF)

S3 Fig The immunogenicity of NPs and Ag was evaluated on THP-1 derived macrophages (left) and immature dendritic cells (right). The cells were incubated for 24h with LPS (1 μg/mL), empty NPs (i.e without Ag, 15 μg/mL), Ag (5 μg/mL) alone or Ag loaded into NPs (30% weight ratio), and the TNF-α, IL-1ß, IL-6 and IL-12p40 secretions were measured by ELISA. Results represent mean ± SEM of 3 independent experiments. Statistical analyses were made by one-way ANOVA * p < 0.05, ** p < 0.01, *** p < 0.001 **** p <0.0001.
(TIF)

S4 Fig (TIF)
