# Supplementary material for: Starch-based NP act as antigen delivery systems without immunomodulating effect
Source: PLoS One. 2022 Jul 29;17(7):e0272234. doi: 10.1371/journal.pone.0272234 (PMC9337643; doi:10.1371/journal.pone.0272234)
Supplement: S1 Methods — (DOCX) [file pone.0272234.s001.docx]

The human monocytic cell line THP-1 was purchased from ATCC (United Kingdom) .

Human monocytic THP-1 cells were maintained in RPMI 1640, supplemented with 10 % heat-inactivated FCS, 100 U/mL Penicillin, 100 mg/mL streptomycin and 1 % (v/v) L-glutamine at 37°C in a humidified 5 % CO2 atmosphere.

The differentiation of THP-1 into a M0 macrophage-like (THP-1 Mφ) adherent phenotype was performed by adding 40 ng/ml PMA to the culture media for 48 h, followed by a further 24 h rest in fresh media with serum. The differentiation of THP-1 into immature dendritic cells (THP-1 iDC) was performed by adding 1500 IU/mL rhIL-4 and rhGM-CSF to the culture media for 5 days, and a medium renewal was performed every 2 days with fresh cytokine-supplemented medium.

For the NP endocytosis studies, they were then treated for 15 to 60 min with 9 µg of DiI-labeled NPs. For antigen delivery study, they were treated for 180 min with 3 µg Ag-FITC, alone or encapsulated in NPL and NPL·NR, and in 500 µL of fresh medium.

Cells were harvested with TrypLE™ Express, collected by centrifugation and diluted in PBS (without calcium or magnesium) before cytometry analysis on an Attune^TM^ NxT (ThermoFisher Scientific). Trypan blue (40 µg/mL) was added to quench FITC extracellular fluorescence. Isolated cells were selected by their size and cellular complexity with a gating on SSC-A:FSC-A then SSC-A:SSC-H. Analyses were made on 7,500 individual cells and the results are expressed as the mean ± sd of at least 3 independent experiments.

For immunogenicity studies, THP-1 differentiated cells were seeded in 96-well plates at a density of 10^4^ cells per well. They were then treated for 24 h, in 250 µL, under different stimulation conditions: RPMI (as negative control), 1µg/mL LPS (as positive control), empty 15 µg/mL NPL or NPL·NR (*i.e* without Ag), 5 µg/mL Ag alone and Ag encapsulated in NPs (5 µg/mL Ag and 15 µg/mL NP). Cell culture supernatants were collected, and cytokine secretions were evaluated with commercial ELISA kits according to the manufacturers’ instructions.

Figure S2

*Figure S 1 :* Ag *was labeled with FITC and loaded in NPL and NPL·NR*. *Its endocytosis on THP-1 differentiated macrophage (A) and immature dendritic cells (B) was evaluated by flow cytometry.* *The results represent the mean ± SEM of at least 3 independent experiments, and the statistical analysis were made by two-way ANOVA tests. * p < 0.05.*

Figure S3

*Figure S 2 : The immunogenicity of NPs and Ag was evaluated on THP-1 derived macrophages (left) and immature dendritic cells (right). The cells were incubated for 24h with LPS (1 µg/mL), empty NPs (i.e without Ag, 15 µg/mL), Ag (5 µg/mL) alone or Ag loaded into NPs (30% weight ratio), and the TNF-α, IL-1ß, IL-6 and IL-12p40 secretions were measured by ELISA. Results represent mean ± SEM of 3 independent experiments. Statistical analyses were made by one-way ANOVA * p < 0.05, ** p < 0.01, *** p < 0.001 **** p <0.0001*.
