# Supplementary material for: Starch-based NP act as antigen delivery systems without immunomodulating effect
Source: PLoS One. 2022 Jul 29;17(7):e0272234. doi: 10.1371/journal.pone.0272234 (PMC9337643; doi:10.1371/journal.pone.0272234)
Supplement: S1 Table — Results represent the mean ± SEM of at least ten independent measurements, made on three independent batches. (DOCX) [file pone.0272234.s002.docx]

|  | Hydrodynamic diameter (nm) | PDI | Z-potential (mV) |
| --- | --- | --- | --- |
| **NP^+^** | 33 ± 2 | 0.26 | + 36 ± 2 |
| **NP^+^·NR** | 17 ± 3 | 0.19 | + 32 ± 2 |
