# Supplementary figures and images for: Starch-based NP act as antigen delivery systems without immunomodulating effect
Source: PLoS One. 2022 Jul 29;17(7):e0272234. doi: 10.1371/journal.pone.0272234 (PMC9337643; doi:10.1371/journal.pone.0272234)

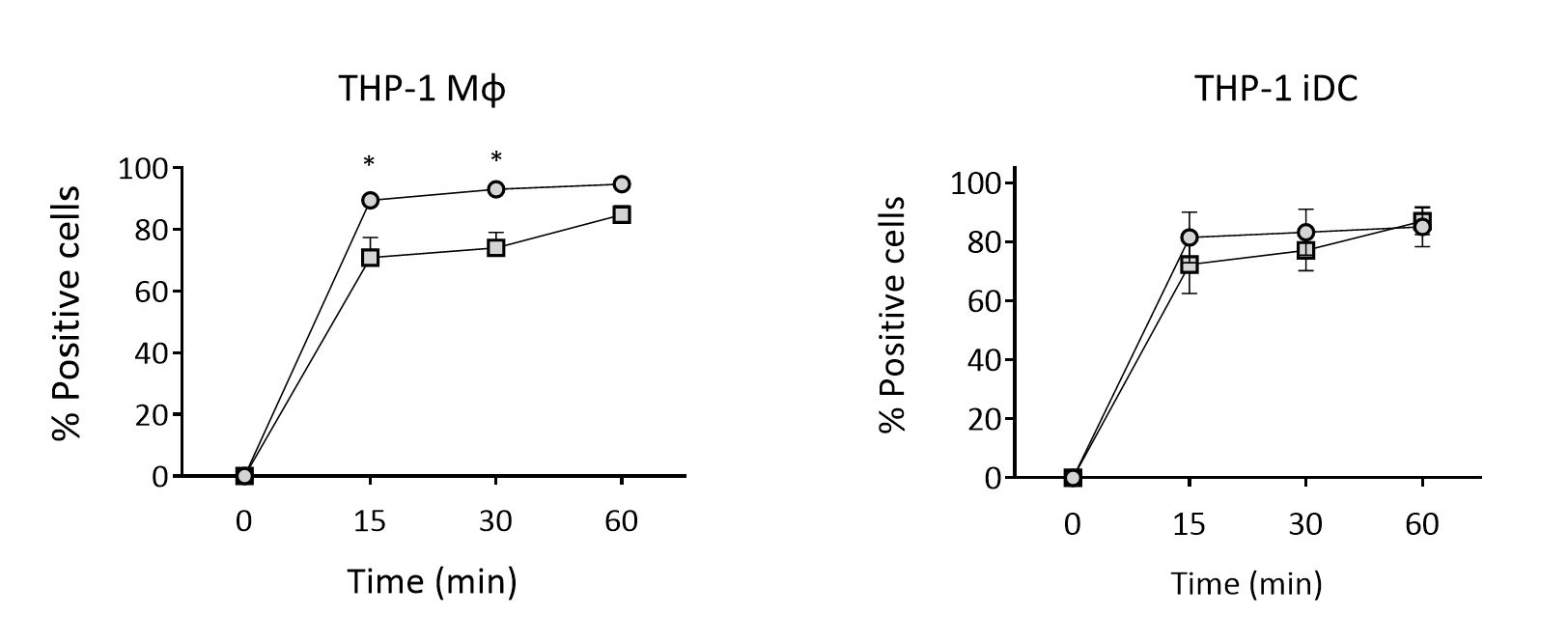

Supplement: S1 Fig — NPs-DiI endocytosis on THP-1 differentiated macrophage (left) and immature dendritic cells (right) cells was evaluated by flow cytometry. The results represent the mean ± SEM of at least 3 independent experiments, and the statistical analysis were made by two-way ANOVA. * p < 0.05. (TIF) [file pone.0272234.s003.tif]

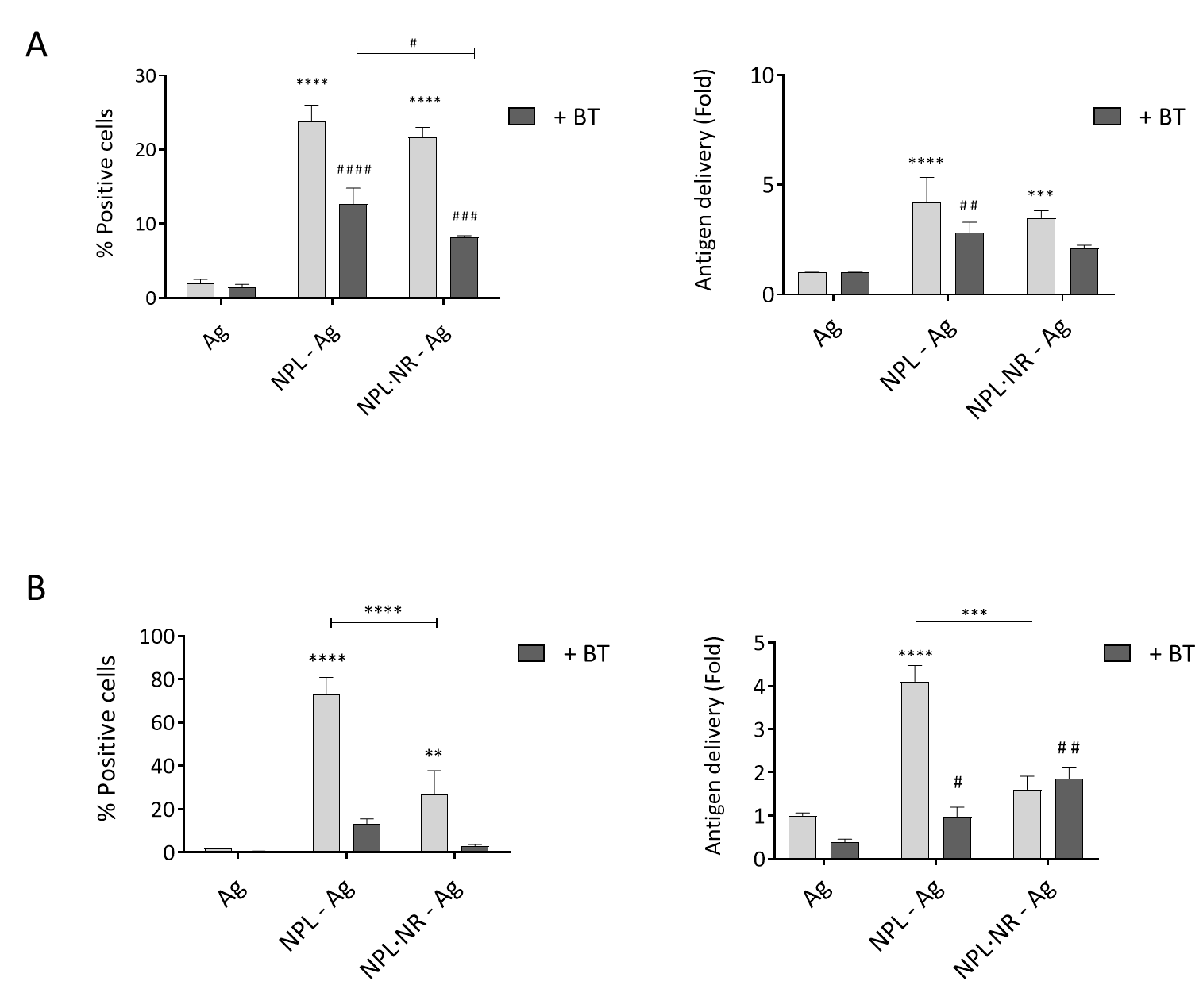

Supplement: S2 Fig — Its endocytosis on THP-1 differentiated macrophage (A) and immature dendritic cells (B) was evaluated by flow cytometry. The results represent the mean ± SEM of at least 3 independent experiments, and the statistical analysis were made by two-way ANOVA tests. * p < 0.05. (TIF) [file pone.0272234.s004.tif]

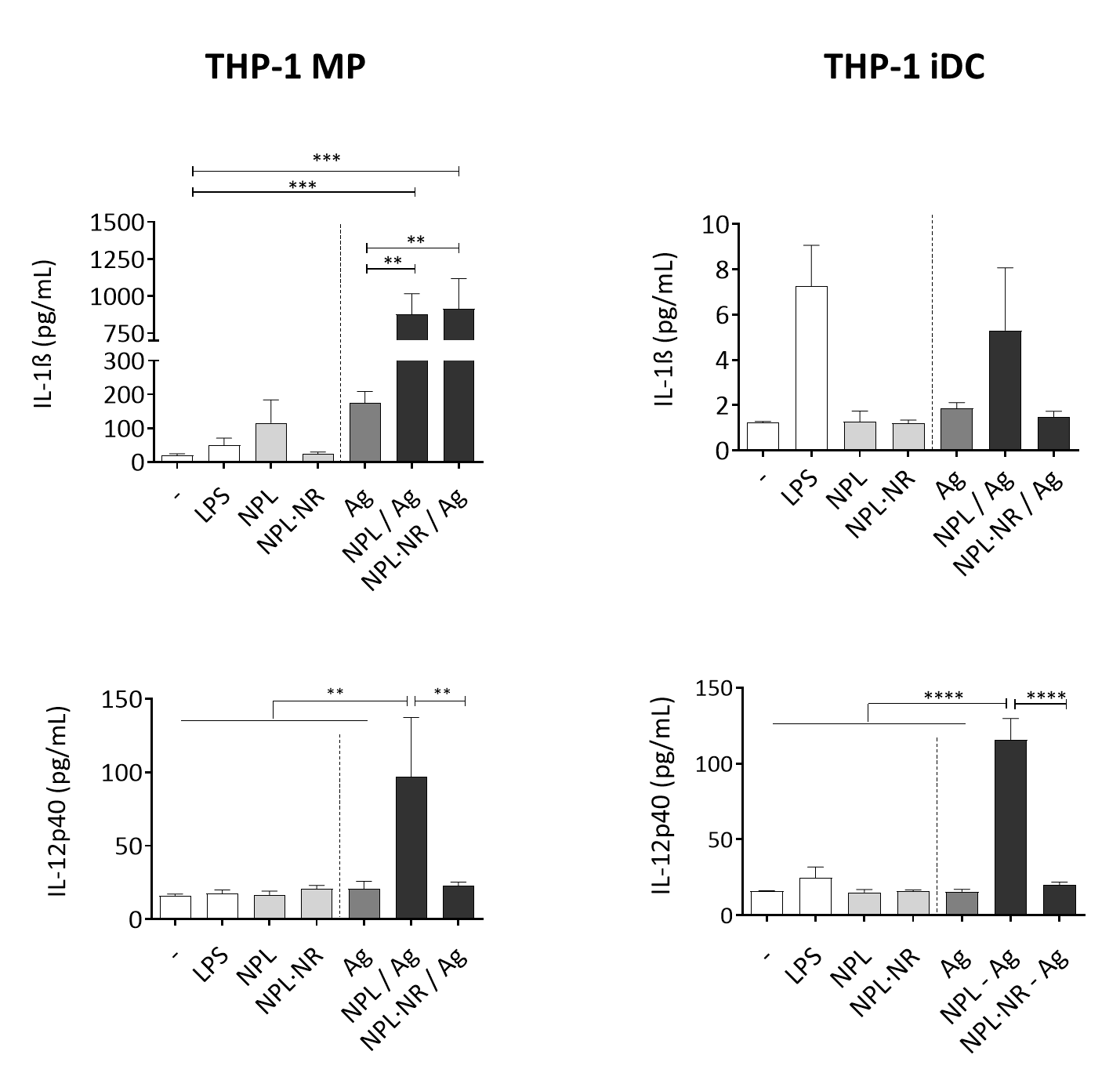

Supplement: S3 Fig — The immunogenicity of NPs and Ag was evaluated on THP-1 derived macrophages (left) and immature dendritic cells (right). The cells were incubated for 24h with LPS (1 μg/mL), empty NPs (i.e without Ag, 15 μg/mL), Ag (5 μg/mL) alone or Ag loaded into NPs (30% weight ratio), and the TNF-α, IL-1ß, IL-6 and IL-12p40 secretions were measured by ELISA. Results represent mean ± SEM of 3 independent experiments. Statistical analyses were made by one-way ANOVA * p < 0.05, ** p < 0.01, *** p < 0.001 **** p <0.0001. (TIF) [file pone.0272234.s005.tif]

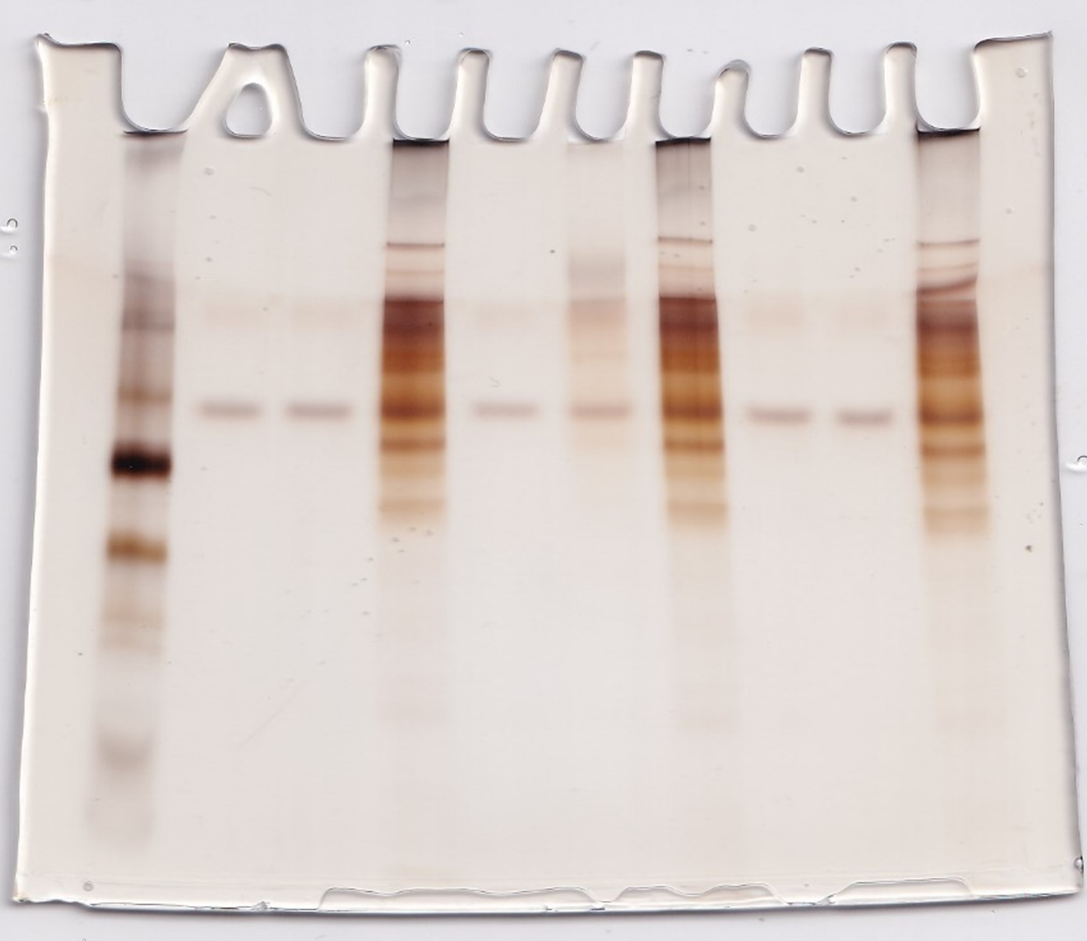

Supplement: S4 Fig — (TIF) [file pone.0272234.s006.tif]
